# The provision of abortion in Australia: service delivery as a bioethical concern

**DOI:** 10.1007/s40592-024-00215-0

**Published:** 2024-09-09

**Authors:** Nathan Emmerich

**Affiliations:** School of Medicine and Psychology, Australian National University, Canberra, Australia

**Keywords:** Abortion, Medical abortion, Surgical abortion, Termination of pregnancy, General practice, Medical education, Specialist medical training

## Abstract

Despite significant progress in the legalization and decriminalization of abortion in Australia over the past decade or more recent research and government reports have made it clear that problems with the provision of services remain. This essay examines such issues and sets forth the view that such issues can and should be seen as (bio)ethical concerns. Whilst conscientious objection—the right to opt-out of provision on the basis of clear ethical reservations—is a legally and morally permissible stance that healthcare professionals can adopt, this does not mean those working in healthcare can simply elect not to be providers absent a clear ethical rationale. Furthermore, simple non-provision would seem to contravene the basic tenants of medical professionalism as well as the oft raised claims of the healthcare professions to put the needs of patients first. Recognizing that much of the progress that has been made over the past three decades can be attributed to the efforts of dedicated healthcare professionals who have dedicated their careers to meeting the profession’s collective responsibilities in this area of women’s health and reproductive healthcare, this paper frames the matter as a collective ethical lapse on the part of healthcare professionals, the healthcare professions and those involved in the management of healthcare institutions. Whilst also acknowledging that a range of complex factors have led to the present situation, that a variety of steps need to be taken to ensure the proper delivery of services that are comprehensive, and that there has been an absence of critical commentary and analysis of this topic by bioethicists, I conclude that there is a need to (re)assess the provision of abortion in Australia at all levels of service delivery and for the healthcare professions and healthcare professionals to take lead in doing so. That this ought to be done is clearly implied by the healthcare profession’s longstanding commitment to prioritizing the needs of patient over their own interests.

## Introduction

For the most part, philosophical concerns regarding the moral status of the fetus and the scope and relevance of autonomy have been the central preoccupation of bioethical discussion of abortion. The clinical or administrative detail of its provision has tended to be seen as a secondary concern and has arguably been relatively neglected. Within philosophical bioethics there seems to be an assumption that legally available public services will be obtainable to those who wish to access them, meaning the relevant healthcare institutions will seek to meet demand for a legal medical service and do so in a proactive manner. However, it is becoming increasingly clear that the provision of abortion in Australia—and, it should be noted, many other jurisdictions, many of which might also be perceived as relatively progressive—leave much to be desired (Baird 2023). Whilst acknowledging that there are a range of factors that impact the provision of abortion in Australia, this essay focuses on the degree to which healthcare professionals, the healthcare professions, and all of those involved in the provision of healthcare and the management of its delivery might not only be considered responsible for some of the issues that women face when seeking to terminate a pregnancy but also positioned to respond.

As such, whilst I do not deny that factors beyond the control of those involved in provision and delivery of services—such as the availability of funding via the Medicare system for example—are also relevant to the circumstance I seek to critique, I nevertheless take the view that those involved could do more to improve service provision, as well as its organization and management in Australia. Indeed, in some cases this can be achieved by ceasing to engage in actions that arguably obstruct the provision of services as well as taking both individual and institutional responsibility for establishing and improving organizational structures and referral pathways whilst also seeking to actively manage the consequences of healthcare professionals ability to conscientiously object (Keogh et al. 2019b; Heino et al. 2013). As this suggests, the healthcare professions and healthcare professionals, particularly medical doctors, have a great deal of power when it comes to the organization, management and provision of healthcare. At least in part, this results from the fact that they present themselves as placing the interests of patients above their own.^1^ What such a commitment might mean is, of course, complex. It does not mean that healthcare professionals must be entirely self-sacrificing and nor does it mean that they must always do as the patient demands (Sokol 2011). Nevertheless, many of the issues that are discussed in what follows seem to be in clear conflict with this fundamental commitment to patients and meeting their needs. Equally, there are broader implications. It would be a mistake to limit the way we understand this commitment to the relationships doctors have to individual patients. For example, the institutions of the professions also present themselves as acting in the collective interests of patients and not just themselves or their members. As such, this essay can be seen as an extended call for all those (who should be) involved in the delivery and provision of abortion services in Australia—something which includes CEO’s of hospitals, managers, consultants in obstetrics and gynecology, and those in general practice as well as professional organizations—to consider if they, and their hospitals, departments and practices, are discharging their responsibilities when it comes to meeting the needs of Australian women seeking to terminate a pregnancy.

This essay is, then, not concerned with the ethics of abortion per se but with *the ethics of provision* in a broad sense. The permissibility of abortion as defined by the law in the relevant jurisdictions is taken for granted. Furthermore, underlying the view set forth in this paper is a supposition regarding the relationship between those things that are provided as (part of a) public service(s) and those who are *served* by those services and therefore seek to access them. In essence the citizens of a particular state^2^—which, regardless of whether it does so directly or indirectly, should be considered the provider of any service rightly termed *public*—should be able to access those services if and when the relevant criteria for doing so obtains.^3^ The implication is that whilst no one institution and no one public servant (which is what healthcare professionals who are employed in public healthcare services effectively are) is obligated to provide the full range of services that falls within the scope of a particular public service, the institutions that constitute the service as a whole must do so.^4^ This point has significant implications for the provision of abortion by public healthcare services and those that manage them.

By way of an example, and one that reflects something of this paper’s concerns, consider the provision of abortion prior to the 9th week (or 64th day) of pregnancy. This can be accomplished via medical or surgical means^5^ and the only relevant criteria that a patient needs to meet in order to be legally entitled to an abortion at this stage is, quite simply, no longer wishing to be pregnant.^6^ However, not only is it the case that many Australian women encounter obstacles when seeking to access an abortion in the early stages of a pregnancy, it is not uncommon for them to be informed about one option, surgical or medical, and not both.^7^ It seems that some providers are routinely omitting the fact that that there is a choice of interventions. Whilst this is generally because only one of the options is immediately available,^8^ this practice clearly conflicts with the ethical principle of informed consent. It also contravenes established law and professional guidelines that govern medical practice in Australia.

It is hard to imagine that this practice would be tolerated in any other area of healthcare. As we shall see it is nevertheless indicative of the kind of flaws that exist within the provision and delivery of abortion services within Australia and, in a similar manner, many other jurisdictions. The primary aim of this essay is to reiterate such matters as ethical concerns and as lapses that call into question the degree to which the healthcare professions are in fact committed to the normative principles they commonly espouse, such as the idea that the patient’s interests are placed above their own. This work is deeply indebted to a range of social scientific and historical research that has brought to light the various issues that are discussed (Baird 2015, 2023; Keogh et al. 2019b, 2021; Newton et al. 2016; Keogh, Croy, Keogh et al. 2019a, b; Costa, Russell, and Carrette 2010; de Costa and Black 2021; Ogden et al. 2021; Sifris and Penovic 2021; Subasinghe et al. 2021; Newton et al. 2016; F. M. Doran and Hornibrook 2016; F. Doran and Hornibrook 2014; LaRoche, Wynn, and Foster 2020; Dawson et al. 2017; Shankar et al. 2017). However, before turning to such matters it is worth giving a brief account of the clinical details relating to the clinical provision of abortion in the Australian context.

## Abortion from a clinical perspective

When it comes to a discussion of ethics in the delivery and provision of abortion in Australia a basic grasp of currently clinical practice is important. Understanding what is being referred to by terms like medical abortion, surgical abortion, vacuum aspiration, dilation and curettage (D&C), as well as what is involved in abortion at late(r) stages of pregnancy, will clearly impact on the way provision is discussed and critiqued. This section offers a brief overview of the various interventions that constitute abortion and some of the regulation pertinent to the Australian context. I do not discuss contraception or emergency contraception.

### Medical abortion

A medical abortion is achieved via combined use of the drugs mifipristone (RU-486) and misoprostol (Mazza et al. 2020). In Australia these are provided under the brand name MS-2 Step, a package which contains the drugs and instructions for their use (Baird 2015). In short, the mifipristone is first taken orally and then, 36 to 48 h, later the misoprostol is taken buccally. In Australia MS-2 Step can be used up to the 9th week of gestation, 63 days after the end of the woman’s last period. Elsewhere the gestational limit for medical abortion has been extended to the 12th week of pregnancy and protocols for its use up to the 15th week of pregnancy exist. Medical abortions can be provided in the community and, partially as a result of arrangements made during the pandemic, the use of telehealth to facilitate provision in Australia is increasingly common. Whilst MS-2 Step has been something that Australian GPs have been able to prescribe since 2013, relatively few have elected to do so or to undertake the relevant training.^9^ A calculation based on the number of active prescriber data published by MS Health and the number of GPs registered with the Medical Board of Australia would indicate that only around 10% of general practitioners are in a position to offer medical abortion (MS Health 2023).^10^

In Australia most medical abortions are provided by dedicated family planning or sexual health clinics. These are often publicly funded but delivered by organizations such as MSI Australia. Private providers also exist. Protocols for the provision of medical abortion generally require an ultrasound to be performed in order that an ectopic pregnancy might be ruled out, a condition that poses a significant risk to the patient’s life and that the drugs for medical abortion cannot resolve.^11^ However, whilst dedicated clinics may have ultrasound machines, few General Practices possess them. Given that many patients would see their general practitioner as the first port of call for healthcare, this represents a barrier to access as it means they must be referred elsewhere before they can use any prescription for MS-2 Step. However, protocols that do not require an ultrasound and instead involve patient’s taking a pregnancy test a few days after the abortion have recently emerged. This approach can be used to better facilitate the provision of medical abortion via telemedicine (Aiken et al. 2021).

In effect a medical abortion induces miscarriage and may therefore result in a particularly heavy period, including significant bleeding and cramping. Whilst medical abortion seems to generally be preferred by women seeing abortions, some may not find it easy to manage or may simply prefer not to have to do so. Thus, whilst protocols for medical abortion might seem to be the first line intervention for patients seeking to terminate a pregnancy, at least some will prefer a surgical approach (Newton et al. 2016). Respect for patient autonomy and the principle of informed consent, as well as professional guidelines and health law in Australia, clearly indicate that patients should be accorded the right to choose between clinically justifiable alternatives. There is therefore a compelling reason to ensure that patients are told about the alternatives to medical abortion and that they are able to reasonably access them.

###  Surgical abortion

Surgical abortion may involve the use of local anesthetic and involves the direct removal of the uterine content either via vacuum aspiration or D&C. In the case of the latter procedure, the uterus is accessed via the vaginal canal and an instrument is passed through the cervix before being manipulated so as to disturb the lining of the womb. Suction can then be applied to remove the content. In some cases, the application of suction—vacuum aspiration—can be sufficient. Surgical abortion can generally take place up to the 16th week of pregnancy and whilst it may be technically feasible to do so, they are not provided in General Practice settings. This is often something that Australian law effectively rules out and, in any event, the clinical requirements mean it would not be financially viable to meet the low level of demand such practices will likely be faced with. As is the case with the majority of medical abortions, it is something that is largely provided by dedicated clinics, such as MSI Australia, at least for the most part. When providing a surgical abortion, it is important that a hospital with obstetric and gynecological department be reasonably proximate in case of an emergency. There are also contraindications which mean a surgical abortion cannot be conducted in a clinic and must take place in a hospital. This includes the patient being significantly overweight or of a particular young age and, relatedly, their anatomy being anomalously small. Regardless, like medical abortion (Goldstone et al. 2017), the safety profile of surgical abortions is well established and complications are exceedingly rare.

### Abortion at late(r) stages of pregnancy

The termination of pregnancy at late(r) gestational stages generally involves the induction of labor, something that is achieved by the use of mifipristone and misoprostol, the same drugs involved in medical abortion. When this is done prior to the 20th week of a pregnancy there is no realistic possibly of the fetus being viable due to insufficient lung development. A range of the fetal conditions that generally motivate termination at late(r) stages of a pregnancy will also mean that stillbirth is a likely outcome or that the fetus will not survive for long. In some cases, however, survival may be a possibility, as might some degree of suffering.^12^ Therefore, there is sometimes a need to perform a feticide. This involves the insertion of a needle through the pregnant woman’s abdomen, into the womb and through the placenta, in order to inject drugs (such as potassium chloride, digoxin, or lidocaine) into the fetus (often directly into its heart) causing the end of its biological life prior to the induction of labor. Like amniocentesis this is a procedure that needs to be guided by ultrasound. Some of those who provide care for pregnant women—and therefore occupy positions in which they may be called upon to provide late(r) term abortions—may not have the experience or skill required to do this. As a result, an additional specialist may need to be involved. In many cases following induction, the fetus will be delivered intact. In others, surgery may be performed, and the fetus will be removed in pieces. Whilst these kinds of intervention generally have good outcomes the nature of the procedures and the risks associated with them mean that the hospital is the only location that an abortion at late(r) stages of pregnancy can be performed. This is something that is also generally encoded in the law of the various Australian jurisdictions, somewhat needlessly as Dwyer et al. (2021) point out.

### Discussion

The provision of abortion is not a singular undertaking. There are a range of interventions that constitute abortion and whilst some are without any real degree of risk there is a need to ensure broader support is available for both patients and providers. As it is the case around the world, the vast majority of abortions take place at earlier stages of pregnancy in Australia. Estimates suggest that around 15–25% of Australian women will have an abortion during their lifetime (Taft et al. 2019; Children by Choice, n.d.). An absence of data means that the precise number of abortions performed in Australia each year is unknown. An attempt to estimate this number suggest that around 88,287 abortions took place in Australia in 2017–18, amounting to around 17 abortions per 1000 women aged 15–44 (Keogh et al. 2021). This was made up of 20,741 medical abortions and 67,546 surgical abortions, the latter number includes the relatively small number of terminations that take place at late(r) stages of pregnancy. Whilst there is little doubt that the number of medical abortions has increased since 2018, with a concomitant lowering of the number of surgical abortions, international data suggest we might expect far more medical abortions to be provided as compared to surgical abortions (Popinchalk and Sedgh 2019).

This would suggest that the ratio of surgical and medical abortions in Australia likely remains skewed and indicates that women are unable to access medical abortion when this is what they would likely prefer. However, it would be wrong to assume that all women prefer medical rather than surgical abortion. It is not only vital to ensure that women are informed about their options but that they are able to make a realistic choice. This means that financial and other costs—such as the distance one must travel to access a particular intervention—should not be disproportionate. At present the majority of medical and surgical abortions that take place in Australia are delivered by dedicated clinics that are publicly funded but exist outside of the established infrastructures. On the one hand, this might be seen as beneficial. Dedicated provision facilitates specialist expertise and, as a result, high quality care. Nevertheless, there is a need to significantly increase provision of medical abortion by General Practitioner’s, not least because existing arrangements reflect and compound exceptionalism about abortion. Furthermore, whilst the Australian Capital Territory (ACT) has recently committed to fully funding abortion services in its jurisdiction (Remeikis 2023), the reliance on dedicated clinics generally means patients face fairly significant financial costs when seeking to terminate a pregnancy in other Australian jurisdictions, an obvious source of ongoing inequity (Subasinghe and Deb 2024). Furthermore, this approach to service provision arguably contributes to the way in which public hospitals are effectively permitted to abdicate their responsibilities when it comes to the provision of such services or to create appropriate referral pathways for patients. Indeed, the fact that public hospitals do not perceive themselves as being required to provide surgical abortion arguably has not insignificant consequences for the way in which late(r) term abortion is provided. Whilst the number of late(r) term abortions is relatively low, there is a need to ensure referral pathways are available and accessible to both healthcare professionals involved in the provision of antenatal care and patients themselves.

## (Bio)ethical concerns in regarding the (non)provision of abortion in Australia

As the quotes which opens this essay, both from Baird’s (2023) recently published history of abortion in Australia, suggest a lack of provision and structural issues in the delivery of services is a—and perhaps *the*—major point of concern in this context at the present time. This was also made clear in two 2023 reports concerning reproductive health services in Australia that were published by the federal and ACT governments (Senate Standing Committees on Community Affairs 2023; Standing Committee on Health and Community Wellbeing 2023). However, what is less clear is the degree to which the ‘accepted practice’ of not having to provide is, in fact, unacceptable or should be seen as such. Two differing aspects of medical practice in Australia permit the non-provision of abortion (or other medical services). First there is the matter of conscientious objection, or the right not to participate in an undertaking one considers to be unethical, something that is discussed further below. Second is the notion that a business, or whoever has operational control of a business, can choose which products or services it wishes to provide. Simply put, if the owner of a corner shop chooses to sell Snickers and not Mars, or if a plumber elects to only install showers and not baths, then it is a matter for themselves. Unless such decisions represent an unlawful form of discrimination, businesses (and private organizations more generally) are free (not) to provide particular products and services as they see fit and in whatever manner they see fit.^13^ Furthermore, they can make such decisions for good reasons (such as the motivation underlying a preference for stocking fairtrade items), for relatively idiosyncratic reasons (such as a personal distaste for a particular item) or they can do so for reasons that are entirely arbitrary. Just because corner shops ordinarily stock a particular item and plumbers generally provide a particular service does not mean any specific corner shop or plumber must do the same.

Given that Australian general practices are, in essence, small businesses, similar points might be said to apply, at least on the face of it. As small businesses they have high degree of independence and if they do not wish to provide a particular service, they are notionally free not to do so. However, whilst this might correctly represent the basic position, this does not mean there is nothing further one might say about it in relation to healthcare, either in general or in the specific case of the significant number of general practices and general practitioners in Australia who are electing not to offer prescriptions for medical abortion. In the first instance, one might note that the provision of medical services and healthcare differs from the provision of goods and services more generally. Healthcare is a public service provided by the state and the Australian government has created an administrative edifice though which it seeks to ensure provision.

As such it is entirely legitimate for the state to be concerned that provision be comprehensive. This does not mean the state should ensure that all general practices (or public hospitals) are willing and able to provide any and all medical services. In at least some areas, including, say, sports medicine, mental health and trans* healthcare, there may be clear rationales for specialization, both in the provision of primary and tertiary care. Nevertheless, ensuring that public services meet existing needs is certainly a state responsibility, and it is therefore legitimate for it to use (or create) levers that enable it to take steps in this regard. For example, if a supplier had a particular interest in ensuring both Snickers and Mars were stocked in every shop it serves, it would be free not to do business with any retailers that refused to sell both or to do so on terms that are disfavorable. Equally, a referral service could refuse to list a plumber because of their decision not to fit baths. The issue is clearly more complex in the case of healthcare. Nevertheless, the state does not have to simply accept non-provision of a particular medical intervention by those it essentially contracts with.^14^

One might further note that as healthcare professionals, general practitioners do not generally present themselves as small business owners with all that might imply. Indeed, as with any other of the healthcare professions, general practitioners hold themselves out as professionals and, in so doing, they affirm the notion that they place the patient’s (health related) interests above their own. Alongside the evident expertise and skill involved in the provision of healthcare, such commitments are part of the reason why the healthcare professions exercise a great deal of autonomy over the nature of their practice. It is also why they enjoy a significant degree of respect, power and authority within modern society. However, if such claims are to be made then it seems appropriate to consider their implications. The claim that the healthcare professions put patients first and prioritize their needs above their own, does not, of course, mean that healthcare professionals are required to do exactly as an individual patient might wish them to. It is certainly accurate to say that healthcare professionals cannot be and should not be compelled to act in a way that they do not consider clinically justified. Equally, however, healthcare professionals are not able to determine the needs of their patients without reference to them. As such, it would seem inappropriate for a particular general practice not to provide a particular service without giving due consideration to the consequences of that decision.

At minimum, then, an *arbitrary* decision not to offer medical abortion seems contrary to the basic commitments of professionalism, not least because if it is arbitrary then the consequences would seem not to have been properly considered. Similarly, an *idiosyncratic* decision not to provide medical abortion would also seem questionable at best. The least one might expect of the healthcare professional who puts the needs of patients first is that such idiosyncratic preferences will not be imposed upon them unnecessarily. Of course, the possibility of a reasoned decision not to provide medical abortion remains. Leaving the idea of conscientious objection to one side, at least for the time being, there may be a range of reasons might motivate non-provision. However, it is worth considering if they constitute good reasons in the light of the overarching commitment to patients that results from the requisite commitment to medical and healthcare professionalism. Indeed, in this context, one might think that a concern for professionalism should be what guides decisions about service provision.

In the first instance there may be clinical reasons for the non-provision of medical abortion. Whilst it is demonstrably safe intervention there is a need to consider the problems that can nevertheless arise and to ensure that an appropriate response can be provided. In essence, however, this is to point out little more than the fact that provision of medical abortion entails more than simply writing a prescription for patients seeking to terminate a pregnancy. As noted, depending on the protocol being followed, an ultrasound to rule out ectopic pregnancy may be required and in rare cases attendance at a hospital may be needed to complete the removal of uterine content. As such, general practitioners need the support of local institutions of they are to provide medical abortion to their patients. This is also true of many other procedures and interventions, something which indicates that there is also good reason to expect the required support to be available. Nevertheless, there are reports of this kind of support being arbitrarily limited, withheld or only begrudgingly offered by tertiary institutions or those employed within them.

Such practices are subject to similar critiques as those currently being levelled at general practice. By way of an example consider the comments made by a general practitioner participating in research into the (non)provision of abortion in Australia who, on the basis of their prior experiences, expressed concern that specialist obstructions and gynecologists at their local hospital would refuse to provide support if and when required to do so and implicitly indicated that providing the required ultrasounds was a favor that they were disinclined to do (Dawson et al. 2017). Providing an ultrasound is, of course, not a favor for the general practitioner. It is a clinical service the patient requires as part of their treatment. As is caring for a patient’s who is experiencing an incomplete medical termination. Once again, if this were this any other area of medicine, one can hardly imagine healthcare professionals refusing or expressing reluctance to provide care in this way. Equally, ensuring that appropriate arrangements are in place should be a matter of establishing the basic infrastructure(s) of healthcare provision. This is a matter of appropriate referral pathways that can facilitate the required degree of mutual coordination between primary and tertiary care, something that is not uncommon. The failure to put these structures in place, and thereby appropriately support general practitioners, seems unique to this area of healthcare and is, at best, something of a lapse on the part of those managing tertiary medical institutions and working within specialist departments of obstetrics and gynecology. At worst it represents an abdication of professional responsibility.

The idea that provision is more than simply writing prescriptions points to a broader issue in the context of primary care. The fact is that too few general practitioners, only around 10%, are trained in the provision of medical abortion. Until recently those who wished to provide medical abortion in had to complete a short online course offered by MSI Australia and then become a registered prescriber of MS-2 Step with the Therapeutic Goods Agency (TGA).^15^ That the unwarranted additional regulation of those who prescribe MS-2 Step in Australia has now been rescinded to is to be welcomed. Nevertheless, the need to offer and undertake training remains, and the course provided by MSI Australia continues to be available. There is, however, some question as to whether such training is entirely sufficient. This is reflected by the fact some of those who provide medical abortion have sought to establish community of practice groups so as to provide support each other as well as those with less experience. Again, such developments are to be welcomed as they address current needs. Nevertheless, the core curriculum for specialist general practice education is the appropriate place to deliver such training. This is something that is not currently the case.^16^ Thus, whilst lacking the clinical competence needed to prescribe MS-2 Step safely is good reason not to do so at the present time, it simply raises questions regarding the need to become competent to do so. If general practitioners do in fact put their patients first, then undertaking the training required to meet their needs seems a relatively minimal requirement.

One final reason that some general practitioners have offered when responding to researchers seeking to understand why they elect not to provide medical abortions is worth considering. Whilst significant progress in the legalization and decriminalization of abortion has recently been made in all Australian states, something that indicates the broad acceptance of its legitimacy on the part of the general public, opposition remains. As a result, abortion can still be subject to judgement and stigma. This is something that extends to providers and not just to those who have abortions. Some general practitioners do not provide medical abortion as they do not wish to be stigmatized for doing so in the local community. This seems to be a particular issue in rural and remote locations, something that compounds the issue of access to such services in such areas. It also seems to be connected to concerns about becoming the only—or one of very few—local providers, something that may not only result in being stigmatized but in having one’s practice become increasingly defined by medical abortion rather than the variation of general practice.^17^

One might, of course, have some sympathy for these kinds of concerns. Nevertheless, particularly in areas where service provision is acknowledged to be insufficient, the claim to put patients first should outweigh them. Equally, one might also note that the structure of this issue reflects that of a prisoner’s dilemma. It is a function of the fact that the majority, if not all, of the general practices and general practitioners in a particular area are electing not to provide the service in question. If a majority were to become providers the (impact of the) stigma of doing so would, at the very least, be distributed and arguably decreased. Equally, there would be little chance of an individual provider’s practice becoming defined by patients seeking to terminate their pregnancy if all, or the majority, of local practitioners were also providers.

### Conscientious objection

In this context, the notion that healthcare professionals can conscientiously object to abortion and therefore opt out of its to the provision is an important one. The notion of conscientious objection exists within and beyond healthcare and reflects the idea that no individual should be compelled to act in a way that they consider to be morally wrong or unethical. There is a great deal of bioethical literature which discusses its application to healthcare and how it should be implemented. Some consider it to be in conflict with the tenants of professionalism and would see the healthcare professionals right to conscientiously object eliminated (Savulescu 2006; Schüklenk and Smalling 2017). Others adopt a more absolute perspectives and would permit healthcare professionals a great deal of leeway when it comes to configuring their practice in accordance with their moral conscience (Oderberg 2019). However, a majority of commentators adopt what might be called a compromise position that allows individuals to opt out of provision but circumscribes the way in which this ought to be done (Card 2020; McLeod 2020; Lynch 2010). The following sketch reflects the consensus around what constitutes a conscientious objection and the need for compromise.

For the most part we are free to act in accordance with the dictates of our moral conscience. If one considers it morally wrong to eat meat as doing so supports industrial farming and causes suffering to millions of animals, one is free to act on one’s beliefs. This does not, however, mean morally motivated vegetarians or vegans are conscientious objectors, they are simply exercising their moral agency as we all do. Conscientious objection is more specific, and it arises when someone is expected to act in a way that they consider to be morally wrong. Thus, it is only when a pacifist is subject to a military draft that they can be considered a conscientious objector. It is also important to note that those who conscientiously object to serving in the military are not permitted to do because they (politically) object to a specific war, even if they consider it not to be morally justified in the specific case.^18^ Rather, conscientious objectors are required to have a universal objection to war or the use of violence and must therefore object on the basis of an absolute moral commitment to pacifism or, at least, a universal rejection of war as a morally permissible undertaking.

In healthcare the question of conscientious objection generally arises in relation to abortion, as well as reproductive services more generally, and assisted dying. It is reasonably clear that those who wish to conscientiously object to being involved in the provision of abortion must consider it absolutely or universally morally wrong to end the life of a human fetus. However, there is a plurality of moral positions that one might adopt with regard to ending the lives of fetuses or, perhaps more accurately, with regard to when the moral value of the fetus might obtain as well as if and when it might be negated or outweighed by the other moral values, priorities or concerns. For current purpose two perspectives are worth considering. First, it might be that full moral status attached to human being at the very beginning of biological life, conception, and that (virtually) all abortions are therefore morally wrong.^19^ Second, it might be that no moral status attaches to the human life until sentience begins to emerge, something that is generally though to occur sometime after the 20th week of pregnancy.^20^

With these moral perspectives in mind consider the fact that, in all likelihood, general practitioners will only be involved in the provision of medical abortion, something that can only be offered to patients who have been pregnant for less than 9 weeks in Australia (and up to 12 weeks in some other jurisdictions). It would therefore seem that those who conscientiously object to providing a medical abortion must adopt the first view mentioned above, that it is wrong to terminate a pregnancy from the point of conception onwards.^17^ In contrast, general practitioners who take the second of the views mentioned above and consider abortion to become wrong when fetal sentience emerges can likely provide medical abortions to their patients, safe in the knowledge that they will almost certainly never be directly involved with surgical abortions or those that take place at late(r) gestational ages.

Similarly, those whose clinical practice means they may be expected to provide abortions at late(r) stages of pregnancy might have more nuanced views than simply universal acceptance or rejection of abortion. Furthermore, they may wish to consider the legal position in the relevant jurisdiction(s) and what it might imply for clinical practice. The law often sets forth criteria that must be met if an abortion is to be performed past a certain gestational stage. However, not only do such criteria vary some jurisdictions do not limit late(r) term abortions. Thus, one can imagine that some healthcare professionals working in the relevant area may not be morally comfortable providing abortions in all legally permitted circumstances. It may be that they should consider conscientiously objecting to provision in general, and therefore opt out of any involvement. This approach differs significantly from that established in relation to those who would conscientiously object to war. Here individuals are required to reject violence on a universal or absolute basis if they are to be considered conscientious objectors. Suggesting that healthcare professionals can opt out of being involved in the provision of abortion when they object to some, but not all, cases is to advance a pragmatic stance, one that results from the fact that healthcare professionals should not put patients in a position where they are required to meet their personal moral criteria if they are to access the (public) service(s) they provide.

Many of the issues discussed in the previous section have emerged from research into conscientious objection and the non-provision of abortion services in Australia. However, a conscientious objection is meant to result from a sincerely held religious or moral belief.^21^ The reasons discussed in the prior section—such as stigmatization and concerns about one’s practice being defined by abortion—are not moral claims. As such, they cannot form the basis of an appeal to the notion of conscientious objection, the purposes of which is to preserve the moral integrity of individual healthcare professionals. This suggests that whilst many of those who opt-out of providing abortion in Australia consider themselves to be conscientious objectors they are at best mistaken and would be better described as convenient objectors (Davis, Haining, and Keogh 2022). This suggests that there is a widespread misunderstanding of the right to conscientiously object amongst healthcare professionals. This is somewhat understandable. Whilst existing regulations are clear about the need for an underlying moral basis of the any claim to conscientiously object, healthcare professionals are not required to express or justify that views when they seek to do so. As such, when implicitly coupled with the fact that general practices and practitioners are essentially small businesses and free to act as such, the idea that the right to ‘conscientiously object’ means it is permissible to simply opt out of provision seems to have taken hold. It is, however, false perspective. Those who would conscientiously object ought to have clear moral reasons for doing so. Furthermore, the arguments presented the previous section suggest that opting out of provision on the basis of a convenient objection conflicts with professionalism in general and the commitment to meeting the needs of patients in particular.

## Conclusion

As noted, the perspective set forth in this paper is very much indebted to research undertaken from historical, social and scientific perspectives. Such studies tend to focus on the complexities of the issue and the variety of factors that have led to the present circumstances. This includes the complex history of abortion law as well as the increased level of regulation mifipristone and misoprostol (MS-2 Step) has been subject too. It also includes: the way abortion—and sexual and reproductive health services more generally—have been funded; the structure and organization of healthcare and the way in which providers are dependent on those who work in other institutions; the way in which education and training has (not) been provided; and the difficulties some providers (perceive themselves to) face in their local communities, particularly when based in rural and remote areas. Recognition of this complexity militates against or, at least, disrupts the idea that there is a simple moral failure of the healthcare professionals and healthcare professionals to meet their collective and individual responsibilities the failure regarding the proper provision of abortion services. It does not, however, preclude the kind of ethical analysis this paper has sought to offer.

As Baird (2023) has convincingly demonstrated, the history of abortion provision in Australia has been a matter of the majority avoiding the issue, and whilst a minority have actively obstructed the provision of abortion, others have devoted themselves to ensuring the availability of services whilst also seeking to make progress in regard removing obstacles to broader delivery. As a result, the situation we see today—where Australia arguably leads the world in the decriminalization and liberalization of laws pertaining to abortion—has in no small degree been brought about thanks to the work of relatively few healthcare professionals as well as a variety of allies, not least grassroots feminist organizations. At the present time further progress is dependent on a greater number of healthcare professionals taking on responsibility and seeking to ensuring abortion services are delivered in a more comprehensive manner and in accordance with the fundamental principles that guide good medical practice as well as those espoused by the healthcare professions more generally.

Whilst no one can be considered responsible for the entirety of the picture I have advanced, a great number of healthcare professionals are in a position to respond to at least some of the concerns that have been outlined. With the exception of those who have serious moral reason to conscientiously object, general practitioners should consider it their duty to ensure they can provide patients with medical abortions. Local hospitals should consider it mandatory to support local providers as required. Those involved in the training of relevant specialists—including general practitioners, obstetrics and gynecology, sexual health and family planning—and who are therefore able to influence established curricula should work to ensure the provision of abortion is considered a basic competency for those they train. In addition, the Royal Colleges should give some thought as to the needs of their members in this regard and how appropriate training might be made available to them.

Finally, bioethicists might give some thought as to the role they have played in this context. Bioethicists have certainly been involved in the legalization and decriminalization of abortion in Australia. Nevertheless, it is not clear that we have made a central contribution to such developments and nor does it seem to be the case that have we have raised concerns about the inadequate provision of services or other ethically troubling practices, such as TRCs.^22^ Bioethics is often critiqued for the way it has become resident ‘in the belly of the medical whale’ (Cooter 2010; Rosenberg 1999, p. 38) and, in effect, become part of medical culture, with a high degree of acceptance of its perspectives and hierarchies being the inevitable result. Yet there is something of a catch-22 here. If it is to make a positive contribution to the (ongoing) ethical debates which arise in regards patient care and professional regulation, it is important for bioethics to be closely connected to and, often times, situated within healthcare and the healthcare professions. Equally, it also remains important for bioethics to maintain an appropriate degree of distance if it is to articulate ethical perspectives that are critical of accepted practice and the way that things are current done. In regards the provision of abortion in Australia, it is not clear that we have fulfilled our function in this regard. It is therefore to be hoped that we can use our positions help those who would seek improve the way services are currently being delivered.

## Data Availability

There is no data associated with this research.

## Footnotes

1 The idea that medical doctors and healthcare professionals should put patients first is commonplace. The Declaration of Geneva states that “[t]he health and well-being of my patient will be my first consideration” (Parsa-Parsi 2017) and the Australian Medical Association’s Position Statement on Medical Professionalism directly refers to and endorses this commitment (AMA 2015). A great number of other statements by professional medical bodies do similarly as do academic analysis of medical professionalism (Swick 2000; General Medical Council (Great Britain) 2013; Medical Professionalism Project 2002).

2 I use the term state to both the nation of Australia and its federated states and territories.

3 If indeed any relevant criteria exist, over and above the individuals desire to avail themselves of it.

4 My point is obviously not that any and all public services must be available to those who need to use them as and when they need to do so. When providing a public service like healthcare it is entirely legitimate to, for example, make decisions about their location with regard to, say, the distribution of the population, the level of need or demand, the exigencies of specialization, and the financial costs involved. Thus, the fact that a particular type of neurosurgery may only be available in one specialist unit across Australia is not the issue. Rather the concern is with ensuring that those who require such surgeries are not restricted from accessing it arbitrarily and, concomitantly, with the need for local services to meet their responsibilities when it comes to facilitating such access.

5 See next section for a brief sketch of the difference between a medical and surgical abortion.

6 The points being made in respect of this issue—i.e. the fact that the only criteria relevant to the provision of abortion during the first trimester of pregnancy in Australia is the individual’s decision to have a termination—can be transposed to jurisdictions, such as the ACT, where there are no criteria which restrict late(r) term abortion. Whilst healthcare professionals may routinely discuss a patient’s reasons for seeking a termination at late(r) stages of pregnancy no one is compelled to offer a reason. Quite simply, unless established in law or clinically inappropriate, there are no grounds for healthcare professionals to refuse a patient’s request for an abortion. This is something that seems to have occurred in the recent past through the use of so called Termination Review Committees (TRCs) (Baird 2023, p. 211; Woodrow 2003; Black, Douglas, and de Costa 2015) and may still be occurring (Bowman-Smart et al. 2023; Haining et al. 2023). Often operating informally and without clear guidelines or principles for decision-making, TRCs were bodies of healthcare professionals convened to determine whether or not a patient would be permitted to have an abortion. In cases where such committees did not result from a legal mandate, they should be considered highly objectionable examples of medical paternalism. Where such bodies were mandated by law, it is arguably the case that those creating such laws effectively place healthcare professionals in a particularly invidious position. Whilst healthcare professionals may establish their own moral perspectives (see the section discussing conscientious objection below) and opt-out of provision on that basis, this does not mean patient access can be constrained on the basis of such views. Where elected representatives have elected not to establish criteria for access, the moral decisions regarding abortion’s permissibility devolves to the individual who is pregnant. If an abortion is legally permissible and the patient wishes to terminate her pregnancy, then the healthcare professionals have no role in preventing it from taking place and the healthcare system as a whole should consider itself duty bound to ensure provision. TRCs are unethically paternalist bodies which conflict with professionalism.

7 This has occurred both where a surgical abortion can be offered but a medical abortion cannot be, and where a medical abortion can be provided but a surgical abortion cannot.

8 As will become clear, the idea that a medical abortion cannot be offered is commonly due to decision made by healthcare professionals. In effect, and whether explicitly or implicitly, they have *chosen* not to offer it.

9 Until recently those wishing to provide medical abortions to their patients were legally required to complete a 2-hour online course provided by MSI Australia and, in so doing, become registered prescribers with the Therapeutic Goods Agency (TGA) (Australian Associated Press 2023; Mazza 2023). This entailed an additional administrative burden when prescribing MS-2 Step and whilst the removal of this needless obstacle is to be welcomed the need for proper training remains and so the course continues to be available. As a result, the fundamental point remains, far too many GPs are choosing not to provide medical abortion and are therefore electing not to undertake the required training.

10 Or, at least, only around 10% were in a position to do so prior to the change in regulation effected in August 2023 (Australian Associated Press 2023). However, as noted, whilst the need to become a registered prescriber has been removed, the need for training remains. It seems reasonable to assume that there has not been a sudden increase in general practitioners taking the relevant training. Following the change in regulations, it is not clear if the data MS Health provides regarding the number of active prescribers will continue to be made available.

11 Ectopic pregnancies occur when a fertilized ova or conceptus implants in the wall of a fallopian tube and not the lining of the uterus. It is not viable and, if untreated, will result in death. Terming it a pregnancy is arguably a misnomer and the same might be said for the idea that treatment, which commonly involves surgical removal of the fallopian tube, amounts to an abortion.

12 In my view it should be acknowledged that, in jurisdictions that do not require certain criteria to be met if an abortion is to take place at a late(r) stage in pregnancy, some patients accessing late(r) stage abortion do so because they no longer wish to have a child. Such decisions are, of course, exceedingly rare. Nevertheless, they do occur.

13 Of course, whatever is provided must meet applicable standards and the regulations governing such goods or services and their provision.

14 Perhaps the most relevant example in this context is the matter of institutional conscientious objection. Catholic ethos organizations—or corporations—have been, and continue to be, permitted to run public hospitals across Australia. This includes managing the provision of maternity and obstetrics and gynecology whilst refusing to provide abortion, either to those who wish to terminate a pregnancy or to (many of) those who have a clear medical indication for doing so. Whilst they have not done so it would be permissible for state and territory governments to refuse to contract the delivery of public services to organizations that refuse to do so comprehensively. Whilst it is a separate point, I should note that I do not consider institutional conscientious objection to be a philosophical justified notion.

15 For an overview on the political developments that first obstructed the introduction of medical abortion to Australia and subsequently produced this recently disestablished system of overregulation, see Baird (2015; 2023).

16 Indeed, it is arguably the case that this should already be part of general practice education. There is no clear reason why becoming registered providers of MS-2 Step should not have been expected of all those training to be general practitioners since the law changed in 2012.

17 One might also note the underfunding of medical abortion provision, meaning that a general practice or practitioner who provides such services may also be financially disadvantaged as a result. Resolution of this matter may be forthcoming with proposals for the introduction of a Medicare item number for medical abortion.

18 The distinction is between *jus ad bellum*, or what constitutes a just war per se, and *jus in bello*, and what constitutes the just conduct of war in particular (Walzer 2015).

19 Consider cases where a continued pregnancy will result in the death of both the pregnant individual and the fetus. This compares to cases of conjoined twins who will both die if they are not separated. In these circumstances saving one life seems morally preferable to losing two, even if the process of saving one life causes or hastens the death of the other.

20 A range of other moral positions are, of course, defensible. This includes the idea that the fetus lacks any moral status until viability, birth or that that its moral status is minimal until personhood emerges (Chervenak et al. 2024; Steinbock and Menzel 2023, Chap. 7). It also includes the idea that whatever moral status a fetus might have, it is insufficient to countervail a women’s (bodily) autonomy and therefore her preference not to be pregnant. Equally, one might remain agnostic and adopt the view that if there is a moral perspective to be taken then the one that matters is that of the individual who is pregnant.

21 Given the supposition that any religious reason for conscientiously objecting to abortion would also be a moral reason, I use the latter term and neglect the former.

22 My thanks to a reviewer who points out that a team of researchers have recently published two papers resulting from research relating to TRCs in Victoria (Bowman-Smart et al. 2023; Haining et al. 2023). As the authors of these works note, they are the first to examine TRCs in almost two decades.
